# How Perceived Career Advising Initiates Career Orientation of UAS Undergraduates in China: Career Exploration as a Mediator

**DOI:** 10.3390/bs15091208

**Published:** 2025-09-05

**Authors:** Tingting Gao, Guoxing Xu, Tingzhi Han, Jiangshan Sun

**Affiliations:** 1 Faculty of Education, East China Normal University, Shanghai 200062, China; 52264105004@stu.ecnu.edu.cn; 2 School of Education, Jiangnan University, Wuxi 214122, China; 8202406006@jiangnan.edu.cn

**Keywords:** career orientation, career exploration, perceived career advising, undergraduates

## Abstract

In the context of universal higher education and labor market polarization, undergraduates in universities of applied sciences (UAS) face growing challenges in forming career orientation. Drawing on data from a UAS in China (N = 3138), this study examines how perceived career advising influences students’ career orientation. Three key findings emerge: (1) Only perceived perspective advising (PPA) exhibits significant and direct effects on career orientation, underscoring the developmental value of structured guidance. In contrast, perceived emotional advising (PEA) and perceived growth advising (PGA) show no direct effect. (2) Both PPA and PGA are positively associated with career exploration, whereas PEA exhibits a negative association. This suggests that when advising interactions are overly affective, they inadvertently reduce students’ initiative to explore. (3) Career exploration fully mediates the effects of PEA and PGA, while it partially mediates the effect of PPA. This reflects that different types of career advising influence career orientation through distinct mechanisms, with PEA and PGA relying more heavily on exploratory engagement. The cultural and educational context in China shapes how students respond to different types of career advising. This study offers theoretical and practical insights for building career advising systems to actively foster students’ autonomous, cognitively engaged exploration processes.

## 1. Introduction

According to the latest OECD (2025) survey, rapidly growing numbers of students are uncertain about their career plans. The global expansion of higher education and labor market restructuring are jointly shaping how undergraduates form and refine their career orientation (Guo & Xiao, 2024), which is not conceived merely as an employment outcome, but as a process-oriented and adaptable approach to career decision making and planning. Within this context, career advising emerges as a pivotal institutional mechanism, which can guide students in clarifying goals, offers dialogic feedback, and sustains motivation.

In East Asia, China’s universities of applied sciences (UAS), which are the main drivers of universal higher education, face mounting challenges in preparing undergraduates for the labor market. The simultaneous transition from a planned to a market-based economy and rapid technological change have intensified labor market polarization, reducing demand for middle-skilled labor and further constraining employment opportunities for UAS graduates (Yang et al., 2024). These challenges are situated within the broader global trend of higher education expansion. By 2020, 76 countries or regions worldwide had reached universal higher education (UNESCO, 2020), yet expanded access has not automatically improved employment outcomes. On the contrary, it has led to structural mismatches in some contexts such as academic inflation and underemployment (Trow, 2007; Figueiredo et al., 2017). The situation exemplifies how large-scale higher education growth, without targeted institutional interventions to foster UAS undergraduates’ clearer career orientations, can exacerbate uncertainties.

Meanwhile, many UAS undergraduates have experienced academic drift in their pursuit of prestige, gradually weakening their alignment with regional industry needs (Jing & Welch, 2018). Consequently, students often enter the workforce with limited career preparation and unclear employment goals (West, 2000). More critically, UAS undergraduates often possess moderate family cultural capital and limited early exposure to career guidance. Once enrolled, they also encounter limited institutional resources and underdeveloped support systems, which severely undermine the development of clear career orientation (Nießen et al., 2022). Evidence from EU applied universities highlights similar patterns that students drop out due to poor career decision making, low career decidedness, misalignment between aspirations and curricula, and lack of access to internships or apprenticeships (Bargmann et al., 2022). In this context, career advising assumes a distinctive compensatory function, filling informational and experiential gaps while supporting students’ goal formation and vocational clarity.

To address these challenges, universities worldwide have established career centers that integrate assessment, practical activities, and mentoring (Lee & Goh, 2003; Schlesinger et al., 2021). However, in many developing countries, professional career guidance remains nascent. For example, students in Pakistan express urgent and diverse needs for career counseling, yet availability remains extremely limited (Keshf & Khanum, 2021). In response, China has adopted a transitional approach by building a broader, more accessible system of career advising as a more feasible foundation where professional counseling infrastructure is insufficient. The government has mandated that universities implement a comprehensive system integrating career advising with academic learning and extracurricular activities, supported by dedicated personnel (Yang et al., 2024). Furthermore, university counselors are expected to maintain regular contact with students to provide personalized career guidance. This system reflects not only policy initiatives but also China’s cultural values of collectivism and its educational tradition of strong institutional involvement. In some universities, however, advising still focuses mainly on information delivery rather than supporting students’ ongoing developmental processes. This limitation highlights the importance of emphasizing the processual role of career advising, particularly its ability to stimulate career exploration, which serves as a key intermediary in fostering students’ career orientation.

Building on the practical challenges highlighted above, researchers have increasingly examined the role of career advising in supporting students’ career development. Wood et al. (1976), through their classic block experiment, demonstrated that effective learning and development require “tutoring” within the learner’s zone of proximal development (ZPD) from “experts”. They extended this concept into what is now known as instructional scaffolding, which enables individuals to move from inability to independence (Van Der Stuyf, 2002). For students in UAS, developing a clear career orientation is often a novel and challenging task. In this context, career advising functions as a crucial scaffold. Recent studies have confirmed the applicability of this scaffolding mechanism. For instance, Zammitti et al. (2023) demonstrated through experiments that group career counseling could effectively promote psychological resources that help university students build coherent career paths. Furthermore, career advising interventions are foundational to the development of value-driven and self-regulated career orientations (Elgeddawy et al., 2024).

Despite these efforts, several limitations persist in the existing literature on career advising. First, many studies reduce career advising to mere information provision or isolated course modules, failing to capture its deeper function as a developmental support system (Crookston, 1972; Laff, 1994). Second, it tends to overemphasize its direct effects, such as enhancing employability or reducing career anxiety, while giving insufficient attention to the behavioral pathways through which advising influences career orientation, particularly via students’ career exploration (Prescod et al., 2019; Zhang et al., 2023). Clarifying this directional relationship is essential for understanding how advising interventions for undergraduates can be optimally designed. Third, limited attention has been paid to how career advising is adapted to the evolving landscape of universal higher education, especially in developing countries where student populations are increasingly diverse and career goals are often ambiguous. Addressing these gaps is of both theoretical and practical value, as it can deepen our understanding of the developmental role of advising and inform the design of more effective support systems for undergraduates.

Building on this foundation, this study investigates how students’ perceived career advising fosters the formation of career orientation by stimulating their exploratory behavior. Focusing on China’s universities of applied sciences (UAS), it situates the analysis within a context where career advising is institutionally mandated but often procedurally underdeveloped. Perceived career advising is conceptualized as a multidimensional construct encompassing perceived perspective advising, emotional advising, and growth advising. Career exploration is introduced as a mediating variable and refers to participation and reflection in career-related activities. Career orientation is defined here not simply as an employment outcome, but as a process-oriented construct denoting the clarity, stability, and value alignment of students’ career goals. Drawing on rigorously sampled data from UAS in China and using structural equation modeling, this study empirically tests the indirect pathway from career advising to career orientation. In doing so, it advances the literature by unpacking the processual role of advising, and extending developmental advising theory to the underexplored context of massified higher education in developing economies.

## 2. Literature Review and Hypothesis Development

### 2.1. Theoretical Foundation and Framework

The conceptual model of this study is grounded in the Social Cognitive Theory of Career Self-Management (SCT-CSM) proposed by Lent and Brown (2013), which emphasizes individuals’ proactive role in managing their career development across diverse educational and occupational contexts. Within the CSM framework, particular attention is given to students’ perceptions of career advising in forms such as verbal persuasion, emotional encouragement, and resource provision, which influence both career-related behaviors and beliefs (Lent et al., 2000; Lent & Brown, 2013). Therefore, rather than acting solely as a direct predictor of outcomes, career advising in this study is theorized to activate and sustain students’ exploratory behavior, serving as a catalyst for behavioral engagement (Stipanovic et al., 2017).

To contextualize how career advising operates in practice, this study also draws on the concept of instructional scaffolding proposed by Wood et al. (1976) to illustrate how career advising enables students’ transition from career uncertainty to clarity. Scaffolding describes how guidance within a learner’s zone of proximal development enables the transition from uncertainty to competence. In the context of career development, advising serves as a scaffold that helps students navigate complex decision making, sustain motivation, and progressively build clarity in their career direction.

**Figure 1:**
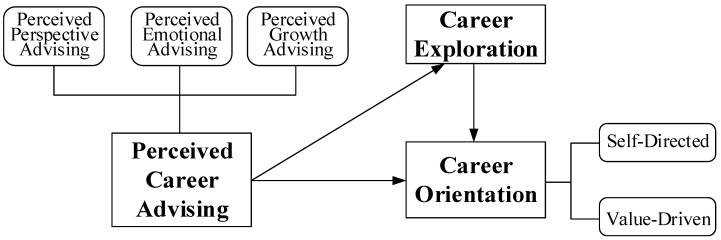
Conceptual farmwork.

Accordingly, the proposed model conceptualizes career advising as contextual scaffolding that stimulates exploration, which in turn fosters the consolidation of career orientation. Within this integrated framework, career exploration functions as the behavioral mechanism linking perceived career advising to career orientation. Exploration involves active participation in, and reflection upon, career-related experiences. Over time, these behaviors consolidate into a career orientation. While exploration and orientation may influence each other, evidence from early career development stages suggests that exploration generally precedes the crystallization of orientation. (See Figure 1).

### 2.2. Career Advising and Perceived Career Advising

The conceptualization of career advising adopted in this study draws from the developmental tradition of academic advising, particularly the seminal work of Crookston (1972), who viewed advising not merely as a transactional process of information delivery, but as a pedagogical and developmental engagement. In his influential article, A Developmental View of Academic Advising as Teaching, Crookston proposed that advising should be understood as an intentional, student-centered, and educational act aimed at promoting students’ self-understanding, decision-making capacity, and long-term life planning. Although Crookston’s focus was originally on academic advising, his underlying framework was deeply intertwined with career development goals, positing advising as a means of empowering students to navigate both educational and vocational pathways. In summary, career advising constitutes a structured developmental intervention designed to support individuals in making informed and meaningful career decisions by providing them with resources, guidance, and reflective engagement opportunities (Hughey et al., 2012).

Distinct from general career guidance or employment counseling, career advising in higher education is increasingly understood as a developmental alliance, which is a sustained, student-centered support system that spans information dissemination, emotional regulation, and motivational scaffolding (Schlosser et al., 2011). Grounded in Social Cognitive Career Theory (SCCT) (Lent et al., 1994), career advising can be conceptualized as a form of contextual support that influences individuals’ career self-efficacy, outcome expectations, and goal-setting behaviors. As a form of social persuasion and vicarious experience, advising helps recalibrate students’ cognitive appraisals of the career environment and enables them to construct future-oriented plans (Zammitti et al., 2023). Drawing more broadly from Bandura’s Social Cognitive Theory (Bandura, 1986), career advising functions as an experience-sharing and self-regulatory mechanism that influences behavior through cognitive reframing and emotional support (Luzzo & Day, 1999).

Building on this view, recent scholarship has further elaborated the developmental advising paradigm, emphasizing the affective, motivational, and future-oriented dimensions of advising interactions (Laff, 1994; Troxel et al., 2021). This developmental stance departs from purely prescriptive or administrative models by foregrounding the advisor–advisee relationship as a space for emotional validation, personal growth, and goal internalization. In the context of Chinese higher education, career advising typically operates within an institutionalized tripartite framework: (a) domain-specific knowledge transmission from faculty members, (b) structured planning and follow-up support from professional advisors, and (c) tacit knowledge exchange within peer groups (Wei et al., 2016). This multidimensional framework fulfills a distinctive psychosocial function, providing scaffolding for students’ career development through cognitive apprenticeship and within communities of practice (Lynch & Lungrin, 2018).

Integrating more recent elaborations, the current study identifies three dimensions of perceived career advising from the perspective of the audience, that is, students: perceived perspective advising, perceived emotional advising, and perceived growth advising. The latter two are part of Crookston’s developmental advising framework.

### 2.3. Career Orientation and Perceived Career Advising

#### 2.3.1. Career Orientation

Career orientation refers to an individual’s cognitive and motivational approach to managing their career in pursuit of subjective success and self-congruence (Hall, 2004). It captures two key psychological dimensions: decision rationality, reflecting the logic, coherence, and deliberative reasoning behind one’s career choices; and directional clarity, denoting the specificity, certainty, and temporal stability of one’s vocational goals (Pesch et al., 2018). These dimensions are theoretically grounded in the concept of the protean career, which emphasizes individual agency, value alignment, and self-direction in navigating nonlinear and dynamic career environments (Hirschi & Koen, 2021). Based on this, Briscoe et al. (2006) developed the first Protean Career Attitudes Scale, which includes two dimensions: self-directed and value-driven orientation.

Among university students, career orientation often emerges as an emergent construct, shaped by personal, social, and contextual influences. While this period allows for identity exploration, students frequently confront bounded rationality and ambiguous goal structures, especially in universal higher education systems (Sargent & Domberger, 2007). A 2019 survey of 663 undergraduates in a mid-ranked UK university found that only 64% reported having a clear career plan by graduation (Quinlan & Corbin, 2023). In China, a longitudinal study revealed that while nearly 70% of third-year undergraduates had a preliminary career direction, most lacked a well-articulated rationale for their choices (Niu & Zheng, 2018). These findings suggest that career orientation is not a static trait, but a malleable developmental outcome influenced by external structuring forces and individual readiness.

#### 2.3.2. The Impact of Perceived Career Advising on Career Orientation

Although much of the existing literature on the formation of career orientation has focused on familial, institutional, and broader sociocultural factors, a growing body of research underscores the significant role of career advising services in enhancing students’ vocational direction awareness and decision-making clarity. Prescod et al. (2019) found that participation in career planning courses and one-on-one advising sessions not only increased students’ engagement in career-related activities but also significantly improved their clarity and confidence in making career decisions. Particularly for undergraduates, faculty members serve as epistemic brokers, acting as intermediaries of knowledge who facilitate the transfer of domain-specific tacit knowledge that bridges academic training and occupational realities (Rivera & Li, 2020). Further evidence underscores that career advising plays a vital role in clarifying students’ future perspectives and cognitive frameworks for decision making. It supports their ability to articulate purposeful goals, weigh options rationally, and construct personally meaningful long-term trajectories (Suryadi et al., 2020).

However, as noted earlier, these studies have not differentiated the specific characteristics of career advising, overlooking the possibility that its content and nature may vary. Some research, of course, has emphasized the importance of examining advisory consultations, mentoring relationships, and longitudinal feedback systems, which together enhance students’ capacity to formulate and pursue coherent developmental pathways (Muceldili et al., 2021; Mujib & Purusa, 2022). In the European context, structured advising programs in Romanian universities were reported to enhance career planning and confidence among over 94% of student participants, with 73% noting improved alignment in their career direction (Staiculescu & Dobrea, 2017). To better determine whether all forms of career advising are not merely informational or administrative, but developmental in nature and capable of shaping students’ career orientation, this study proposes the following hypotheses:

**H1a.**  *Perceived perspective advising positively influences undergraduates’ career orientation.*

**H1b.**  *Perceived emotional advising positively influences undergraduates’ career orientation.*

**H1c.**  *Perceived growth advising positively influences undergraduates’ career orientation.*

### 2.4. Career Exploration and Its Mediational Role

The construct of career exploration traces its conceptual roots to Jordaan’s (1963) operational definition, which characterizes it as goal-oriented activities generating self- and occupational knowledge. Building on this foundation, Super’s (1980) life-span theory posits career exploration as a developmental task through which individuals crystallize their vocational self-concept during emerging adulthood. Within higher education contexts, this process entails systematic appraisal of career-relevant environmental affordances (Flum & Blustein, 2000), with recent empirical work emphasizing students’ agentic engagement (Cheung & Arnold, 2014). Empirical evidence delineates three-tiered benefits of career exploration: (a) socio-cognitive differentiation, involving the evaluation of personal values against occupational stereotypes; (b) agentic capacity building, which enhances self-directed career management competencies; and (c) intrapersonal calibration, referring to the alignment of personal aptitudes with career demands (Storme & Celik, 2018).

The mediating role of career exploration is grounded in Social Cognitive Career Theory (SCCT) and goal-setting theories, which posit that environmental affordances (e.g., advising interventions) influence distal outcomes indirectly via activation of agentic career behaviors (Lent et al., 2000; Locke & Latham, 2002). In this view, as a goal-directed behavior, career exploration is typically stimulated by contextual resources such as structured advising, institutional scaffolding, and relational support (Li et al., 2022). A randomized controlled trial in China revealed that intensive advising interventions significantly improved students’ more proactive career behaviors (Zhang et al., 2023). In particular, career advising activates exploration by reducing decisional paralysis, offering access to occupational information, and encouraging reflective engagement (Peng et al., 2020; Perdrix et al., 2012). Emotional and developmental support from advisors also buffers the cognitive and affective costs of exploration, thereby increasing students’ willingness to invest in exploratory activities. Based on these findings, this study proposes the following hypothesis:

**H2a–c.**  *Perceived career advising (perceived perspective advising/perceived emotional advising/perceived growth advising) positively influences undergraduates’ career exploration.*

Hirschi and Koen (2021) integrate career orientations with career self-management in a dynamic self-regulatory framework. Within this framework, career exploration is conceptualized as a critical behavioral stage through which career orientation is both shaped by action and, once established, guides further exploration. However, existing research on undergraduates has predominantly emphasized career exploration as the antecedent of orientation, as exploration enables students to clarify goals, assess value congruence, and transform abstract intentions into actionable plans (Dozier et al., 2015; Hirschi & Pang, 2023). From this perspective, career exploration serves as a developmental mechanism through which external support is translated into internalized and structural orientation (Gati & Asher, 2001). Applying this logic to China’s UAS context further underscores the appropriateness of treating career exploration as a mediating factor. Given that student resources are often limited in these institutions, effective perceived career advising becomes the critical catalyst for initiating exploratory behavior (Gordon & Steele, 2015). By stimulating students’ engagement in career-related activities and reflection, advising not only encourages exploration but also facilitates the career orientations. Based on these findings, this study proposes the following hypotheses:

**H3.**  *Career exploration positively influences undergraduates’ career orientation.*

**H4a–c.**  *Career exploration mediates the impact of perceived career advising (perceived perspective advising/perceived emotional advising/perceived growth advising) on undergraduates’ career orientation.*

## 3. Methodology

### 3.1. Sampling

After validating the questionnaire through a pilot study, we conducted formal data collection from University S, China, in January 2025. University S was deliberately selected because it holds a median ranking among Chinese universities and is widely recognized as a representative type of UAS, characterized by its focus on application-oriented training, close industry linkages, and a student body that largely pursues engineering and technology disciplines.

As of December 2024, the University S enrolled approximately 18,000 undergraduates across 13 faculties, 11 of which offer undergraduate programs spanning a diverse range of academic disciplines, including engineering and technology, and social sciences. This disciplinary breadth and balanced institutional profile enhance the representativeness of the sample for the UAS sector in China.

Data were collected with the assistance of the Communist Youth League of University S, which distributed an online survey link to all enrolled undergraduates. Participation was voluntary. Stratification was conducted proportionally according to the university’s official enrollment data on gender, grade, and majors, thus aligning the sample with the actual student population structure. To ensure representativeness, the research team conducted targeted secondary invitations to underrepresented groups. Ultimately, valid responses were obtained from 3138 students, representing approximately 20% of the undergraduate population. The final sample distribution in terms of gender, grade, and academic major closely matched the university’s enrollment profile, thereby enhancing the representativeness of the data.

Overall, the sample consisted of 2007 male students (64%) and 1131 female students (36%). Most students (81.89%) were enrolled in engineering and technology-related subjects, while the remaining students pursued majors in public administration, labor and social security, business administration, and other related fields. The distribution of students by grade level was as follows: 1,128 (35.9%), 879 (28%), 668 (21.3%), and 463 (14.8%).

The survey plan and instruments, as well as the overall research protocol, were approved by the institutional ethics review board. Before participation, students received a written consent form detailing the study’s purpose, anonymity assurances, data confidentiality, and potential risks.

### 3.2. Variable Measurement

A questionnaire was designed to investigate undergraduates’ career advising experiences and assess their level of career orientation. The instrument primarily measured perceived career advising, career exploration, and career orientation.

#### 3.2.1. Perceived Career Advising Scale

PCAS measures individual levels of perceived career advising through 17 items divided into three subscales: perceived perspective advising, perceived emotional advising, and perceived growth advising. This construct emphasizes the student-centered nature of advising, recognizing that its impact depends not only on the content delivered but also on how such support is subjectively understood and internalized by students (Bandura, 1997; Lent & Brown, 2013). Importantly, this framework includes both formal and informal advising agents, acknowledging that in resource-constrained institutional settings such as Chinese universities of applied sciences (UAS), effective support may come from peers, teachers, family members, or counselors. The conceptualization of career advising was primarily informed by Crookston’s (1972) developmental advising model, which views advising not as a transactional or administrative task but as an educational dialogue that fosters students’ reflective thinking and goal setting.

The initial 20 items were generated based on a systematic review of prior advising literature, including career development programs and advising handbooks (e.g., Hughey et al., 2012). These items were then refined through expert review by three higher education specialists to ensure content validity and cultural fit. Cultural adaptation was particularly important, as advising practices in Chinese UAS contexts often extend beyond trained professionals to include academic or administrative personnel with varying levels of guidance experience. Sample items include “Helped me identify opportunities for career development”, “Helped me alleviate stress”, and “Made me grow into a better person”. Responses were recorded on a five-point Likert scale from 1 (strongly disagree) to 5 (strongly agree), with higher scores reflecting a greater perceived quality of career advising among undergraduates. Exploratory factor analysis (EFA) validated the scale, and three items with low or ambiguous loadings were removed during the process, resulting in a Cronbach’s alpha value of 0.939 in the formal survey.

#### 3.2.2. Career Orientation Scale

Career orientation was measured using the protean career orientation scale adapted by Borges et al. (2015) for university students, based on Hall’s (1996) conceptual framework. The scale comprises 20 items across two dimensions: self-directed orientation and value-driven orientation. The scale was culturally adapted for the Chinese context and demonstrated satisfactory reliability and validity. Sample items include “I have difficulty identifying opportunities to develop my career” and “I believe that success is much more valuable than a good salary”. Items were rated on a five-point Likert scale ranging from 1 (strongly disagree) to 5 (strongly agree), with higher scores indicating a more rational and clear career orientation among undergraduates.

#### 3.2.3. Career Exploration Scale

To capture the construct more comprehensively, CES includes 8 items and integrated both behavioral exploration and reflective exploration. Career exploration measures were adapted from a subscale of the Career Development Inventory (CDI) (Thompson et al., 1981), which was subsequently modified for career exploration assessment by Lokan (1984). Behavioral items assessed their active engagement in exploratory actions (e.g., examples include “I actively seek out opportunities to participate in career development activities”), while reflective items assessed students’ evaluative awareness of their career development (e.g., “After activities, I realize I need to improve myself)”. Items were rated on a five-point Likert scale ranging from 1 (strongly disagree) to 5 (strongly agree), where higher values indicate a deeper level of career exploration among undergraduates.

### 3.3. Data Analysis Technique

The study variables are classified as predictor and outcome variables; however, their underlying structures and relationships are complex. Therefore, the study employed a variance-based structural equation model (VB-SEM), which is particularly appropriate for modeling multiple latent variables and complex causal relationships, and this approach aims to explore the predictive validity of these relationships. In addition, while maximum likelihood estimation (MLE) is widely adopted in social science research for model construction, the present study employed Mplus 8.3 with the robust maximum likelihood estimator (MLR) to accommodate the non-normal distribution of some variables and ensure robust estimation in the mediation model (Hox et al., 2010).

To capture the hierarchical nature of the outcome construct (Koufteros et al., 2009), a second-order latent factor was incorporated for career orientation (CO) and career exploration (CE). Predictor variables included three dimensions of perceived career advising: perspective advising (PPA), emotional advising (PEA), and growth advising (PGA). Control variables were also included to reduce confounding, comprising gender, grade, major, family economic and cultural background, academic performance, and student leadership experience.

## 4. Data Analysis and Results

### 4.1. Measurement Model Analysis

Prior to conducting structural equation modeling, the reliability and validity of each latent construct were examined. As shown in Table 1, all variables demonstrated exceptionally high Cronbach’s alpha, ranging from 0.921 to 0.967. All exceeding the 0.70 threshold, indicating high internal consistency. Convergent validity, which reflects the degree to which items are correlated with their underlying construct, was evaluated through average variance extracted (AVE) and composite reliability (CR). The AVE values of all constructs exceeded the recommended cutoff of 0.50 (Fornell & Larcker, 1981), thus supporting convergent validity.

Furthermore, discriminant validity was examined using the Fornell–Larcker criterion, which requires that the square root of each construct’s AVE be greater than its correlations with other constructs (Fornell & Larcker, 1981). Discriminant validity was also confirmed, as the square roots of AVE were greater than the inter-construct correlations, indicating adequate separation among constructs (see Table 2). These results demonstrate that the measurement model has good psychometric properties for subsequent structural analyses.

In addition, the results indicated relatively high internal correlations within perceived career advising. Therefore, a thorough evaluation of common method bias (CMB) was performed following the guidelines suggested by Podsakoff et al. Harman’s one-factor approach was applied to determine whether one factor dominated the variance across all items. The unrotated principal component analysis revealed that the largest single factor accounted for 30.01% of the total variance, remaining well under the 50% cutoff.

### 4.2. Path Analysis

According to Bentler (1990), the SEM model fits the data well (CMIN/DF = 4.241, *p* < 0.001, CFI = 0.890, TLI = 0.884, RMSEA = 0.057). Following the standards set by Kline (2011), the CMIN/DF value should typically fall between 3 and 5, with lower values indicating better model fit. In this study, the CMIN/DF value is 4.241, which falls within the acceptable range.

As shown in Table 3, structural path estimates were used to examine the effects of perceived career advising (PCA) on students’ career exploration (CE) and career orientation (CO). PPA had significant direct effects on CO (β = 0.231, *p* < 0.001). In contrast, neither PEA nor PGA showed significant direct effects on either outcome variable.

All three dimensions of PCA significantly predicted CE. PPA and PGA had strong positive effects on CE (β = 0.494 and β = 0.514, respectively; both *p* < 0.001), while PEA had a significant negative effect (β = −0.195, *p* < 0.01). CE, in turn, exerted strong positive effects on CO (β = 0.750, *p* < 0.001).

### 4.3. Mediation Analysis

In the paper by Gunzler et al. (2013), the mediation effects were examined and are summarized in Table 4, and H4 was supported. CE was found to significantly mediate the associations between different dimensions of PCA and CO.

Among the three advising types, PPA exhibited a partial mediation effect on CO. Specifically, the indirect effect of PPA on CO through CE was significant (β = 0.372, *p* < 0.001), and the direct path was also significant (β = 0.231, *p* < 0.001), indicating a stable and complementary relationship. The proportion of mediation reached approximately 61.79%, suggesting that more than half of PPA’s total impact on CO was explained via CE.

In contrast, PEA demonstrated a full mediation effect on CO. While the indirect effects were statistically significant and negative (β = −0.147, *p* < 0.01), the direct effects were nonsignificant, implying that CE fully mediated the influence of PEA.

PGA also showed a complete mediation through CE. Although the direct effects on CO were not statistically significant, the indirect effects were significant and positive (β = 0.387, *p* < 0.001). In these cases, CE not only mediates but explains and reverses the underlying effects, underscoring its central role in connecting developmental advising to CO.

Taken together, these findings indicate that CE functions as a critical mechanism linking PCA to students’ CO. While PPA promotes CO through a dual pathway, PEA and PGA rely more heavily on CE to exert their influence, pointing to the varied psychological routes through which advising shapes undergraduates’ CO.

## 5. Discussion

### 5.1. Only PPA Directly and Positively Influences Career Orientation

The results partially support H1a–c. Among the three types of advising, only PPA demonstrated significant and stable direct effects on CO. It echoes earlier work suggesting that goal clarity and decision-related cognitive inputs are core antecedents of self-determined career planning (Lent & Brown, 2013). In the Chinese context, where higher education is highly structured and influenced by collectivist cultural norms, students tend to rely on concrete guidance and clearly defined pathways to reduce uncertainty in career choices (Sun & Yuen, 2012).

In contrast, PEA and PGA showed no significant direct associations with CO. One explanation could be that emotionally affirming or aspirational conversations, though valuable for trust-building, may fall short of mobilizing students’ planning capacities if they are not supported by actionable strategies or tangible goals (Sampson et al., 2004). These results challenge the assumption that all forms of support foster career development equally (Gati & Asulin-Peretz, 2011). While previous literature has highlighted the motivational benefits of affective support (Bagci, 2018), our findings suggest that if not anchored in instrumental content, such advising may not yield measurable effects on students’ career direction. As Dey and Cruz Vergara (2014), former Vice Provost for Student Affairs at Stanford University, emphasized, new initiatives must be incorporated into existing career services to address evolving student needs.

### 5.2. PPA and PGA Encourage Career Exploration While PEA Hinder It

H2a–c are partially confirmed by the findings, which indicate that PPA and PGA significantly predict students’ engagement in career exploration. This suggests that when students receive advising that offers direction or encourages long-term development, they are more likely to initiate exploration efforts, such as seeking information, reflecting on alternatives, or engaging in internships. This pattern aligns with previous studies on exploratory behavior (Super, 1980; Hirschi, 2020). Developmental advising that provides both structure and encouragement appears particularly effective in facilitating students’ transition from passive contemplation to active planning.

However, PEA exhibited an unexpected negative effect on career exploration. This finding contradicts prior assumptions that emotional support necessarily strengthens students’ engagement. Similar patterns have been observed in counseling research, where emotional reassurance without cognitive structuring may foster dependency and decreased self-efficacy (Lapan et al., 2001). In China, cultural norms emphasizing deference to authority and collectivist expectations may exacerbate this effect, as students could interpret emotional support as external control rather than as a prompt for autonomous action (Davis et al., 2012). Moreover, students with high uncertainty or low confidence may interpret emotional support as a signal of external control (Ryan & Deci, 2000). In short, without sufficient instrumental orientation, perceived emotional advising cannot serve as an effective foundation for exploration.

### 5.3. Career Exploration Mediates the Effects of PPA but the Patterns Differed

Consistent with Hypotheses H3 and H4a–c, CE significantly predicted CO. More importantly, CE mediated the effects of all three types of PPA, but the patterns differed across advising types.

PEA and PGA, which are grounded in developmental framework, were fully mediated by CE. Crookston (1972) and colleagues argued that the core of developmental advising lies in stimulating individuals’ intrinsic motivation, reflection, and self-regulatory capacities. This aligns with Social Cognitive Career Theory (Lent et al., 1994), viewing developmental advising as support that shapes career expectations, operating through exploration. Therefore, the effects of PEA and PGA interventions must operate through observable behaviors to translate into eventual career orientation (Koen et al., 2012). In particular, the estimated mediation proportion of PGA exceeding 100% suggests a suppressor effect. Suppressor effects of this nature have been reported in research on career identity transitions (Briscoe et al., 2012), and suggest that career exploration functions not merely as a pathway but as a reframing mechanism.

In contrast, PPA exhibited partial mediation. This suggests that PPA has both a direct cognitive–structural effect and an indirect developmental effect via career exploration. For example, presenting career paths or job postings enables students to immediately set goals. However, sometimes these resources still require students to engage in exploration to achieve optimal alignment (Chen et al., 2024). Compared with Western contexts, where individualized, student-centered advising and self-directed exploration are often more culturally normative (Schell, 2023), Chinese students may rely more heavily on structured, perspective-oriented input before they feel confident to engage in autonomous exploration. This dual pathway is consistent with career construction theory (Savickas, 2005), emphasizing that both direct guidance and exploratory experience contribute to identity consolidation. These findings underscore the importance of career advising frameworks that not only deliver structured input but also actively cultivate students’ exploratory agency.

## 6. Conclusions and Implications

This study examined how different forms of perceived career advising influence undergraduates’ career orientation, and how these effects are mediated by career exploration. By triggering students’ exploratory engagement, perceived career advising plays an initiating role in shaping career orientation, functioning as a developmental scaffold.

### 6.1. Strengthening Structured PA and Activating the Functions of EA and GA

The results underscore that not all forms of advising contribute equally to students’ career orientation. Research on the causes of unclear career orientation among Chinese undergraduates has largely focused on institutional support, without comparing different support models or distinguishing their effects from students’ individual engagement (Niu & Zheng, 2018). In contrast, our study examines the differential roles of various advising types, finding that only perspective advising (PA) directly promotes career orientation, growth advising (GA) exerts influence through students’ active exploration, and emotional advising (EA) can even have a negative association.

Therefore, any effective career advising system must first ensure the stable implementation of perspective advising. To operationalize this, faculty and staff of UAS can be trained to deliver targeted advising modules that include goal clarification tools, decision-mapping exercises, and labor market literacy sessions. Such structured guidance can help undergraduates construct more rational career orientations by linking self-assessment with objective professional knowledge.

With PA established, the potential of emotional and growth advising can be more fully activated. EA can be redesigned to function not only as reassurance, but as a catalyst for exploration when paired with specific career information. For example, advising sessions can include emotional check-ins followed by values clarification or narrative career construction tasks, which help students transform affective states into motivational energy. Similarly, GA should be scaffolded through structured goal-setting templates, personal development plans, and milestone tracking systems. Advisors can assist students in identifying long-term aspirations and breaking them down into actionable steps (Yang et al., 2024).

In summary, PA provides a factual and logical framework, EA facilitates the internalization of values and emotional reflection, and GA supports long-term planning and actionable pathways. As a result, students develop career orientations that combine rational judgment with value alignment. Practically, this integrated approach mitigates the risks associated with relying on a single advising form, such as cognitive overload or emotional distress, thereby fostering more stable and actionable career orientations.

### 6.2. Build an Exploration-Centered Career Advising System

The findings confirm that career exploration is a key developmental mechanism through which career advising translates into career orientation, which reinforce the assumption of Social Cognitive Career Theory (SCCT). A large body of research across countries has unequivocally acknowledged the crucial role of career exploration in shaping individuals’ career orientation and has sought to enhance the efficiency of exploration (Chen et al., 2024). However, for disadvantaged student groups such as undergraduates in UAS, exploration does not occur spontaneously. Accordingly, career education in universities should prioritize stimulating students’ career exploration.

Practically, universities could build an exploration-centered career advising system. For instance, early-stage advising may focus on broad exposure, such as industry talks, career simulations, or alumni panels. Whereas mid- to late-stage advising can incorporate applied learning opportunities such as guided internships, job shadowing, or service-learning projects. These should be intentionally sequenced with pre-exploration advising sessions and post-exploration reflection modules to ensure students make meaning from experience and update their goals accordingly (Kang, 2023). Through this structured cycle, students are not only encouraged to initiate exploration but are also supported in transforming exploratory experiences into clarified values and concrete goals, which ultimately strengthens their career orientation.

Moreover, consistent with its educational positioning, UAS could embed career exploration into general education curricula, allowing students to investigate real-world problems, conduct informational interviews, and co-construct evolving career identities with peers and mentors (Venson et al., 2016). Such modules not only bridge advising and curriculum, but also reposition exploration as a developmental competency that guides students from academic study to vocational choice in shaping their career orientation.

### 6.3. Limitations and Future Research

While this study provides robust theoretical and empirical contributions, several limitations should be acknowledged. First, the focus on Chinese universities may constrain the generalizability of the findings to other educational and cultural contexts. More importantly, the conclusions are deeply embedded in the specific cultural features of Chinese higher education, such as hierarchical faculty–student relations, collective value orientations, and the influence of family expectations. These cultural characteristics may shape how students perceive and engage with different advising modes, thereby limiting the transferability of the findings to other socio-cultural settings. Future research could incorporate cross-institutional or cross-cultural comparisons.

Second, the cross-sectional design limits the ability to capture dynamic changes in how career advising and exploration interact over time. Future research should employ longitudinal or mixed-method approaches to examine these processes across different academic stages. Furthermore, the study did not incorporate variables reflecting students’ internal psychological characteristics, such as career decision self-efficacy, which may influence both engagement with advising and career orientation outcomes.

Third, some potentially important factors, such as personality traits, prior internship experience, or peer influences, were not controlled. These factors could systematically affect how students respond to different advising types, their engagement in career exploration, and ultimately, their career orientation. Future studies could integrate these variables into more comprehensive models to better understand the conditions under which different advising strategies are most effective.

## Figures and Tables

**Table 1:** Reliability and validity testing of measurement instruments.

Variable	Cronbach’s Alpha	CR	AVE
Perceived perspective advising	0.921	0.924	0.789
Perceived emotional advising	0.961	0.968	0.835
Perceived growth advising	0.952	0.953	0.769
Career exploration	0.967	0.967	0.786
Career orientation	0.958	0.979	0.697

**Table 2:** Descriptive statistics and Fornell–Larcker criterion.

	M	SD	1	2	3	4	5
1. Perceived perspective advising	3.666	0.879	**0.888**				
2. Perceived emotional advising	3.779	0.831	0.873 ***	**0.914**			
3. Perceived growth advising	3.747	0.829	0.875 ***	0.903	**0.877**		
4. Career exploration	3.797	0.850	0.773 ***	0.735 ***	0.767 ***	**0.886**	
5. Career orientation	3.882	0.888	0.709 ***	0.658 ***	0.679 ***	0.815 ***	**0.835**

Note: In the discriminant validity table, the diagonal cells in bold represent the square root of the AVE values, and the lower triangle represents the Pearson correlation coefficients. *** indicates *p* < 0.001.

**Table 3:** Path coefficients analysis.

Path	β	S.E.	C.R.	*p*	Result
H1a: PPA→CO	0.231	0.048	4.959	0.000	Accepted
H1b: PEA→CO	−0.008	0.046	−0.175	0.861	Rejected
H1c: PGA→CO	−0.096	0.056	−1.664	0.096	Rejected
H2a: PPA→CE	0.494	0.051	10.177	0.000	Accepted
H2b: PEA→CE	−0.195	0.062	−3.072	0.002	Accepted
H2c: PGA→CE	0.514	0.068	7.574	0.000	Accepted
H3: CE→CO	0.752	0.031	23.822	0.000	Accepted

Note: PPA = perceived perspective advising, PEA = perceived emotional advising, PGA = perceived growth advising, CE = career exploration, CO = career orientation.

**Table 4:** Mediation test.

Path		Effect Value	S.E.	BC 95% CI		*p*	Proportion of Mediation Effect
				Lower 5%	Upper 5%		
PPA→CE→CO	Indirect effect	0.372	0.039	0.279	0.446	0.000	61.79%
	Direct effect	0.231	0.046	0.114	0.315	0.000	
	Total effect	0.602	0.052	0.481	0.706	0.000	
PEA→CE→CO	Indirect effect	−0.147	0.048	−0.260	−0.065	0.002	94.84%
	Direct effect	−0.008	0.048	−0.127	0.068	0.861	
	Total effect	−0.155	0.066	−0.310	−0.046	0.018	
PGA→CE→CO	Indirect effect	0.387	0.053	0.243	0.465	0.000	132.99%
	Direct effect	−0.096	0.057	−0.239	−0.001	0.095	
	Total effect	0.291	0.072	0.104	0.400	0.000	

## Data Availability

The raw data supporting the conclusions of this article will be made available by the authors on request.
